# Music in business and management studies: a systematic literature review and research agenda

**DOI:** 10.1007/s11301-023-00339-3

**Published:** 2023-03-27

**Authors:** Elia Pizzolitto

**Affiliations:** Department of Business Economics, University G. D’Annunzio – Chieti-Pescara, Viale Pindaro, 42, 65127 Pescara, PE Italy

**Keywords:** Music, Business, Management, Musicians, Arts, Digital revolution, Systematic literature review, Grounded analysis, JM0, JM1, JM2, JM3, JL21, JL26, JZ11

## Abstract

Music is the background of life, representing an international language that connects different cultures. It is also significant with respect to economies, markets, and businesses. The literature in the music field has identified several issues related to the role of digitalization in the revolution of music, the distribution of music products, the management and organization of music events, music marketing strategies, and the position of musicians as entrepreneurs. This paper comprises a systematic literature review of the most recent articles discussing the numerous connections between music, business, and management (2017–2022). Through a rigorous protocol, this research discusses the effects of the digital revolution on the music industry, with particular reference to the persisting oligopoly of major labels and the new business models that integrate music streaming and social networks. The findings show the renaissance and relevance of live music events, the fundamental role of segmentation strategies for managing festivals, and the limited presence of sustainability as a priority during festivals and events management. Furthermore, the literature highlights the relevance of discussions concerning musicians’ identity, especially in light of the complex relationship between the bohemian and the entrepreneurial nature of their profession. This is followed by numerous reflections on future research opportunities, recommending theoretical and empirical in-depth studies of music industry competition, futuristic management philosophies and business models, and the roles of technology, sustainability, and financial elements in fostering artists’ success in the digital era. Finally, the paper discusses business models and strategies for musicians, festivals management, stores, and sustainability.

## Introduction

As Mithen (2009; p 3) states, “To be human is to be musical”. Music is part of most individuals’ daily lives. For example, it plays in the background when people make purchases at stores and eat at restaurants. It is a universal language that helps strangers communicate immediately, that spreads and evokes emotions, and that inspires players, producers, and listeners (Cooke 1990; Hunter and Schellenberg 2010). However, beyond being an artform that can connect people from different cultures (Huron 2001) and with different identities (Mithen 2009), music is also a business.

The worldwide relevance of music can be recognized not only in philosophical discussions but also in statistical data about the industry. Music consumption has grown quickly, particularly since the start of the digital revolution, and this growth seems unlikely to slow down in the future (IFPI 2022). The worldwide music industry has grown significantly in recent years. In fact, it grew from US$14.2 billion in 2014 to US$25.9 billion in 2021, revealing growth of 18.5% in 2021 (IFPI 2022). Streaming now drives the music market, representing 65% of global music market revenues in 2021 (IFPI 2022). This trend is a consequence of the digital revolution, which has been characterized by two phases of development (Koh et al. 2019). The first phase involved physical and digital music record sales. The second phase was the development of streaming, unbundling, and cross-platform services that combined music with other entertainment forms, such as video games, television programs, films, and talent shows (Shen et al. 2019).

Two of the most relevant dimensions of the music industry are as follows: (a) the production and distribution of music through physical and digital support networks, as guided by record companies; and (b) the production and distribution of live music, which is controlled by world-famous artists but is characterized by many minor professional musicians, sound technicians, and other workers. These two dimensions are interconnected. The digital revolution is disruptive, and it has upset traditional capitalist economies, but the world of live music has resisted such changes (Azzellini et al. 2021). In addition to these two major dimensions, the music industry includes a complex and elaborate set of other dimensions. These comprise the conditions of minor musicians and labels; publishing, managing, and marketing; teaching and other educational activities (Thomson 2013); and the conditions of local music and record stores.

In this regard, multiple issues have emerged from the literature, mostly related to the big change that the digital revolution has brought about in the music industry. For example, minor artists and labels have to consider new marketing strategies, and they need to find innovative ways to exploit the easier connections between consumers and their products that technologies allow, despite having limited financial assets (Zhang 2018). Another example involves music events or festivals, which face complex challenges because of the recent Covid-19 pandemic. Innovation in the organization and marketing of these events is focused on the concept of value creation, guided by the idea of festivals as chaotic and unpredictable events in which collaboration and co-creation are critical for the achievement of financial, economic, and social objectives (Werner et al. 2019). Covid-19 and the digital revolution complicated the conditions of local music and record stores, which are more centered on experience than competition and need stable innovation in their marketing strategies (Trabucchi et al. 2017).

Moreover, the growth of the music business has highlighted the complex relationship between musicians’ artistic and entrepreneurial sides. In fact, musicians face cultural barriers to their identification as professionals. As Frederickson and Rooney (1990) noted, an absence of formal requisites to enter the musical profession is one of its most relevant barriers; because of this lack of a need for credentials, many people do not consider music to be a profession. Indeed, as Henry (2013) found, few music students think about teaching music as a future profession. According to Pizzolitto (2021), musicians are reluctant to consider themselves entrepreneurs because of the complex relationship between art and profit. Therefore, the entrepreneurial identity of musicians should be fostered to overcome this obstacle to recognizing music as a profession.

This may be challenging, as the Covid-19 pandemic has adversely affected musicians’ activities. Although the music industry has continued to grow and has not been experienced many negative consequences, there have been numerous (orderly) protests by musicians and sound technicians who feel they have been forgotten by governmental policies. For example, in October 2020, a large group of musicians played in front of the British Parliament to protest a decision to decrease state benefits for freelance workers (Savage 2020). During the same month, musicians and sound technicians peacefully occupied many public squares in Italy to broadcast the slogan *Esistiamo anche noi*, which translates to “We also exist” (Sky Tg24 2020). In November 2020, musicians in Berlin held silent protests against a lockdown (Global Times 2020). Indeed, music around the world has experienced a number of conflicts.

Beyond the disparities in the success of record companies versus professional musicians, there are philosophical complexities connected to an antinomy between the artistic nature of music and the capitalistic context in which it is developed (e.g., see Bridson et al. 2017; Haynes and Marshall 2017). Musicians and record producers also face strategic issues concerning the distribution of their music. Waldfogel (2017) defined the current era as a golden age for music listening, yet musicians and producers experience dilemmas every time they intend to launch a product. One dilemma relates to the many opportunities available because of the digital revolution; for example, one can take advantage of digital platforms, streaming, and physical support networks. However, the increase in options demands more in-depth strategic planning. Thus, the philosophical significance of music to individuals, its relevance to people’s lives, and its importance to the economy are at odds with the research conditions of the field.

While attempts to map the literature on music research have been numerous and of high quality, they have mainly concentrated on the effects of music in the workplace (e.g., Landay and Harms 2019) or music education research. For instance, in 2004, volume 32 (issue 3) of *Psychology of Music* focused on mapping music education research in single national contexts (e.g., Welch et al. 2004; Gruhn 2004). Moreover, in 2006, Roulston published a methodological paper that incentivized qualitative research in the music field. Therefore, while existing research has discussed content relevant to the field, there is no literature review on the recent development of music in business and management studies, including topics such as digitalization, the conditions of the industry and competition, the management of music events, innovations, sustainability, and the complex position of musicians after the digital revolution. Consequently, this article addresses the research question “How does recent literature debate music in business and management studies?”.

The next section provides details on the methodology used in this literature review. In particular, it gives a detailed explanation of the procedure used to gather data and analyze the content of articles. Following this is a descriptive analysis of the literature sample, including the journals, author affiliations, and methods of the articles, and an analysis of the contents of the articles. The next section studies the themes that emerged in the articles. Finally, future research opportunities, managerial implications, and a general discussion of the results are presented in the conclusion.

## Methodology

A systematic literature review (SLR) was chosen as the methodology for this study for two main reasons. First, the research questions concern a specific field (i.e., music in business studies). Second, an SLR can ensure a greater degree of objectivity compared with other kinds of literature reviews (e.g., narrative). It can also ensure reproducibility and limit biases in article selection and interpretation (Denyer and Tranfield 2009; Post et al. 2020). This study used a method described by Wolfswinkel et al. (2013), where contents of articles are analyzed using a grounded theory approach. This method ensured that the analysis would not be based on any prejudices. Moreover, grounded theory provides the advantage of developing a theoretical framework in the absence of a specific background (Corbin and Strauss 1990; Strauss and Corbin 1997). Finally, the method published by Wolfswinkel et al. (2013) exhibited no article selection differences compared with other SLR methodologies (e.g., see Denyer and Tranfield 2009; Post et al. 2020). Therefore, the method met all the requirements for conducting an SLR as objectively as possible.

### The employed protocol

**Fig. 1:**
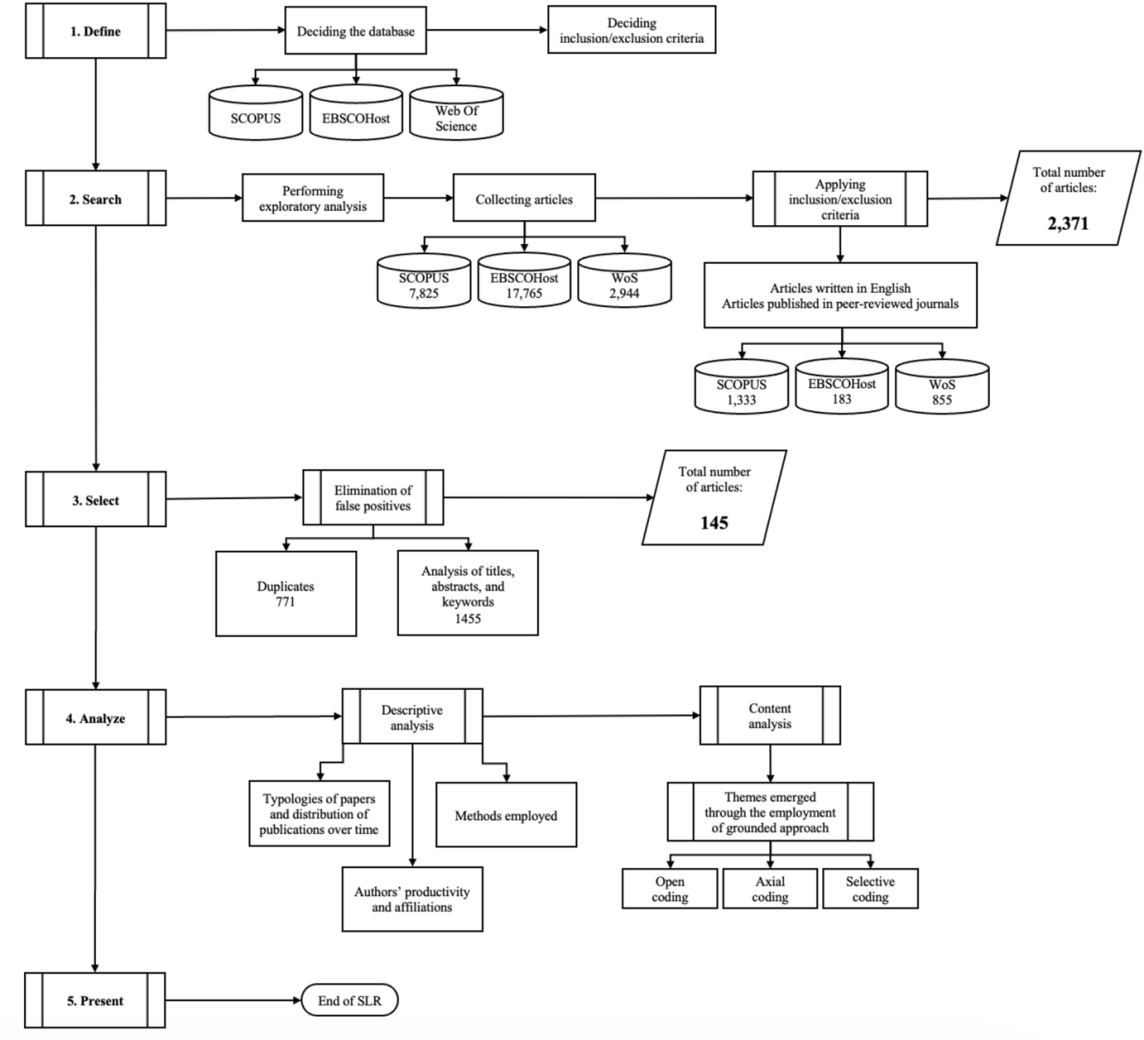
Phases of SLR employed protocol

The method included the five following phases: define, search, select, analyze, and present (Fig. 1). During the first phase, the database and the inclusion/exclusion criteria for the study were determined. Similar to most SLRs (e.g., see Vrontis and Christofi 2019), only articles that were written in English and that had been published in peer-reviewed journals were included in the sample. To ensure that articles of the highest quality would be included, only those listed in the SCOPUS, EBSCOHost, and Web of Science databases were considered. Finally, specific keywords were used to search the three databases and were applied to titles, abstracts, and keywords of the articles. The keywords were *music AND *(*business OR management*).

The second phase consisted of the search for articles. This began with several exploratory analyses to ensure that all relevant literature would be included. The research commenced with searching SCOPUS, and 7825 results were retrieved. The results were limited to peer-reviewed articles written in English, which narrowed the list to 3764 results. As the focus was on recent literature regarding music in business studies, the publication years were then restricted to 2017–2022, leaving 1333 results.

The same steps were followed in searching EBSCOHost. The initial dataset included 17,765 results, and after the limitations were applied, 183 articles remained. The steps were repeated in searching Web of Science, resulting in an initial sample of 2944 results. This number decreased to 855 after the limitations were applied.

During the third phase, the 2371 results were refined, and 771 duplicates were eliminated. After screening the titles, abstracts, keywords, and contents, 1444 false positives were eliminated. The final dataset comprised 145 articles.

The last two steps of the procedure were “analyze” and “present.” The next section presents the descriptive and content analyses of the articles (i.e., the results of these steps). Analyzing the articles included determining the type of paper, the distribution of publications over time, the authors’ productivity and affiliations, and the methods employed in the empirical articles. The contents of the articles were analyzed using a grounded theory approach, during which four themes emerged.

### Inclusion and exclusion criteria

This section details the methods applied when including or excluding articles. To facilitate the selection of articles, an Excel file with 19 columns and 145 rows was created. More than 2700 cells were filled in with the following information for each paper: ID number, DOI, authors, title, publication year, source title, number of citations, source (i.e., SCOPUS, EBSCOHost, or Web of Science), paper type (i.e., empirical, conceptual, review), methodology (i.e., qualitative or quantitative), methods employed, sample size, type of statistical units; authors’ provenience; and theories cited. For reasons of space, the table cannot be shown here, but it is available upon request.

In addition, a column was devoted to the reason for including or excluding a paper. The objective was to consider only articles that explicitly referred to the interconnections between music and business or management issues. The process started with evaluating the titles, abstracts, and keywords to gain an understanding of the articles’ contents. If this evaluation was insufficient, the articles were read for a better evaluation.

To ensure transparency in the selection process, as suggested by Denyer and Tranfield (2009), some examples are considered. One article that was retrieved from the Scopus database was the article *Neumatic singing in Thai popular singing, 1925–1967* by Inkhong et al. (2020) because it included the keywords used for the initial extraction. The article discussed solutions to problems related to incorrect pronunciation using neumatic singing. Therefore, it was excluded. Another article, *Quality management of music education in modern kindergarten: Educational expectations of families* by Boyakova (2018) talked about understanding and improving the quality of children’s music education and made no reference to business or management studies. Therefore, it too was excluded.

Examples of articles that were included are as follows. The article *Digital music and the “death of the long tail”* by Coelho and Mendes (2019) discussed the impact of digital distribution in the music market, concentrating on the dualism between the long tail theory and the superstar effect theory. Schediwy et al. (2018)’s article *Do bohemian and entrepreneurial career identities compete or cohere?* discussed the identity of musicians. The article *The role of stakeholders in shifting environmental practices of music festivals in British Columbia, Canada* by Hazel and Mason (2020) discussed festival management. These three articles were relevant to our study and were therefore included in our review.

### Grounded analysis of the articles’ contents

The grounded theory approach employed in this review followed a certain protocol. The 145 papers included in the sample were divided into subsamples of five randomly selected papers using Excel. Open coding was used to label the subsamples with codes to identify concepts and enable comparisons of the articles, including information such as the affiliated intuitions, the methodologies used, and the theoretical in-depth analyses. The final aim is to conceptualize the most relevant aspects and identify categories and subcategories of common elements.

Axial coding was then employed to make connections between the codes and facilitate further comparison of data emerging from the articles and theoretical and methodological frameworks in the various subsamples. The final aim was to build a systematically integrated network of concepts.

Finally, selective coding was employed to conceptualize and improve the results of the previous analyses. The aim was to achieve a well-integrated theoretical reasoning that can be used to simplify and enrich the reflections on the studied phenomenon.

**Fig. 2:**
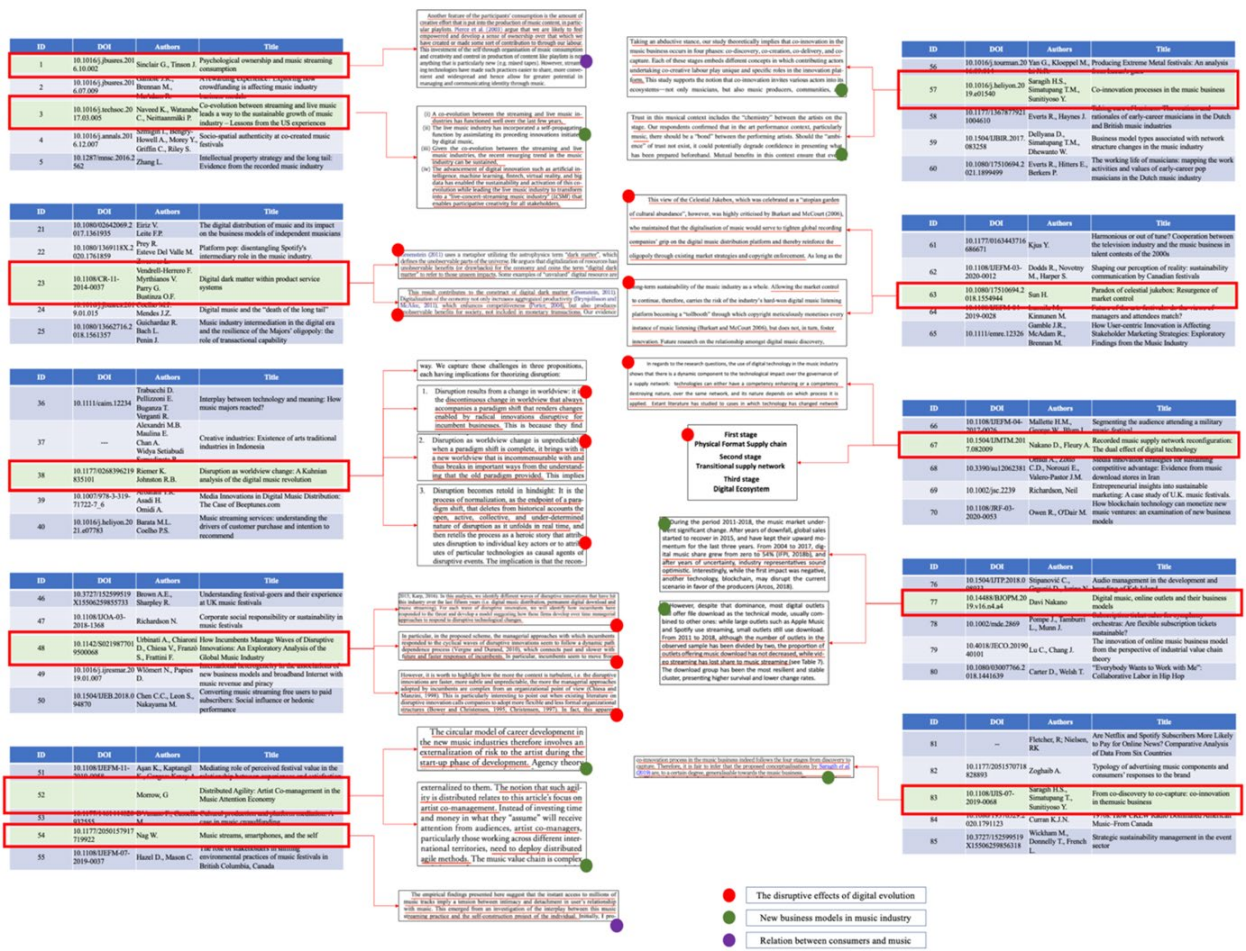
An example of grounded methodology applied in this research (colour figure online)

Figure 2 shows an example of how subthemes emerged during the analysis. The blue tables correspond to random samples of five articles. The red oblongs represent the most relevant articles. The black squares represent (a very limited number of) critical codes (open coding). The violet, red, and green circles represent the results of conceptual connections among the codes (axial coding); the interpretation of these connections is shown at the bottom of the figure.

### Limitations

This literature review tries to develop a complete picture of the most relevant research in music management and business. The aim is to establish a starting point for future in-depth analysis and gathering of future research in this area. Nevertheless, it is not exempt from limitations. First, the methodology, database searches, and analyses were performed by one author. Although the selected method (grounded theory method; see Wolfswinkel et al. 2013) was chosen for its ability to reduce objectivity, even in the content analysis of the selected articles, there might be limitations in the application of inclusion and exclusion criteria considering the high number of articles.

Second, this review employed specific databases to ensure the quality of papers extracted. However, this method inevitably excluded books, book chapters, grey literature, and other sources of information that could be relevant to the topic and in terms of triangulating information and enriching the results of the analysis. Therefore, future research should include the object of the analysis and more sources of information.

Finally, this review limits the time span to recent years. Although SLRs should include a limited number of articles to concentrate on a very specific and relevant research question, the considerable body of knowledge in terms of music management and business suggests that a more comprehensive viewpoint can be considered. Nevertheless, in case future research would be interested in enlarging the time span of the analysis, the amount of articles that would emerge would be studied using quantitative methods, such as meta-analysis or bibliometric analysis.

## Descriptive analysis

**Fig. 3:**
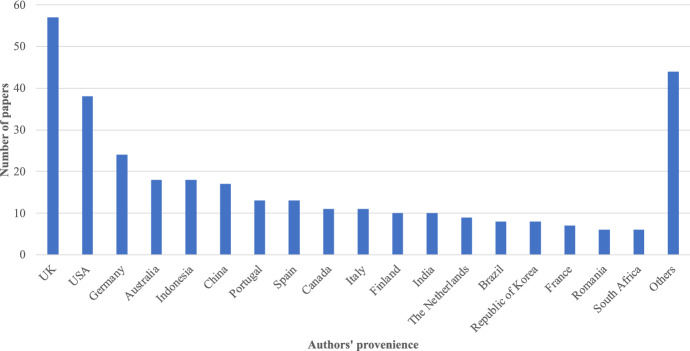
Authors’ provenience

This section presents the descriptive analysis of the selected literature. The analysis focuses on three aspects: (1) the authors’ provenience (Fig. 3), (2) the types of papers, and (3) the methods employed in the empirical studies.

**Fig. 4:**
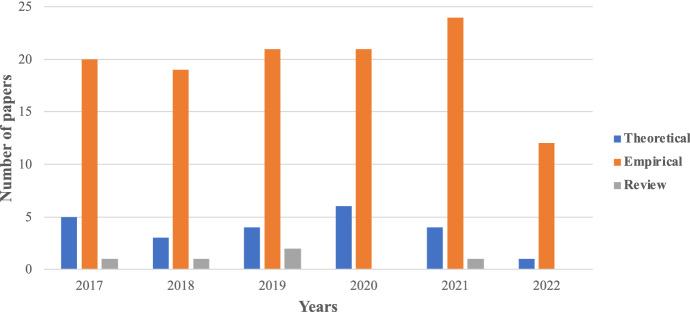
Papers’ typology per year

**Fig. 5:**
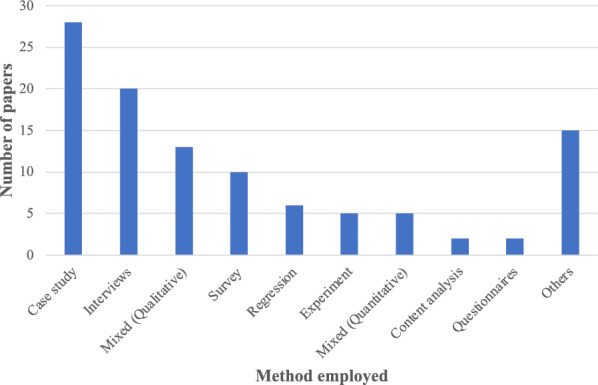
Methods employed in the selected articles

The UK and the USA led in terms of the number of relevant articles published in the period under study with 28.96% of the articles. There is a worldwide interest among scientists in terms of music research in business studies. Even though empirical articles are the most common in the selected dataset, theoretical articles and literature reviews are present in each of the five years considered. Excluding the year 2022, in which theoretical articles represent less than 8% of the contributions, one fifth of the dataset from 2017 to 2021 consists of theoretical articles and literature reviews. Therefore, there is considerable interest in debating conceptual issues in this field. Qualitative research—particularly case studies (26.42% of the dataset)—is the most common methodology used for performing research in the field. Interviews were also commonly used in the extracted contributions (18.87%). Therefore, it is possible that qualitative frameworks of analysis are the best way to gather and evaluate data in this market (Figs. 4 and 5).

## Content analysis

**Fig. 6:**
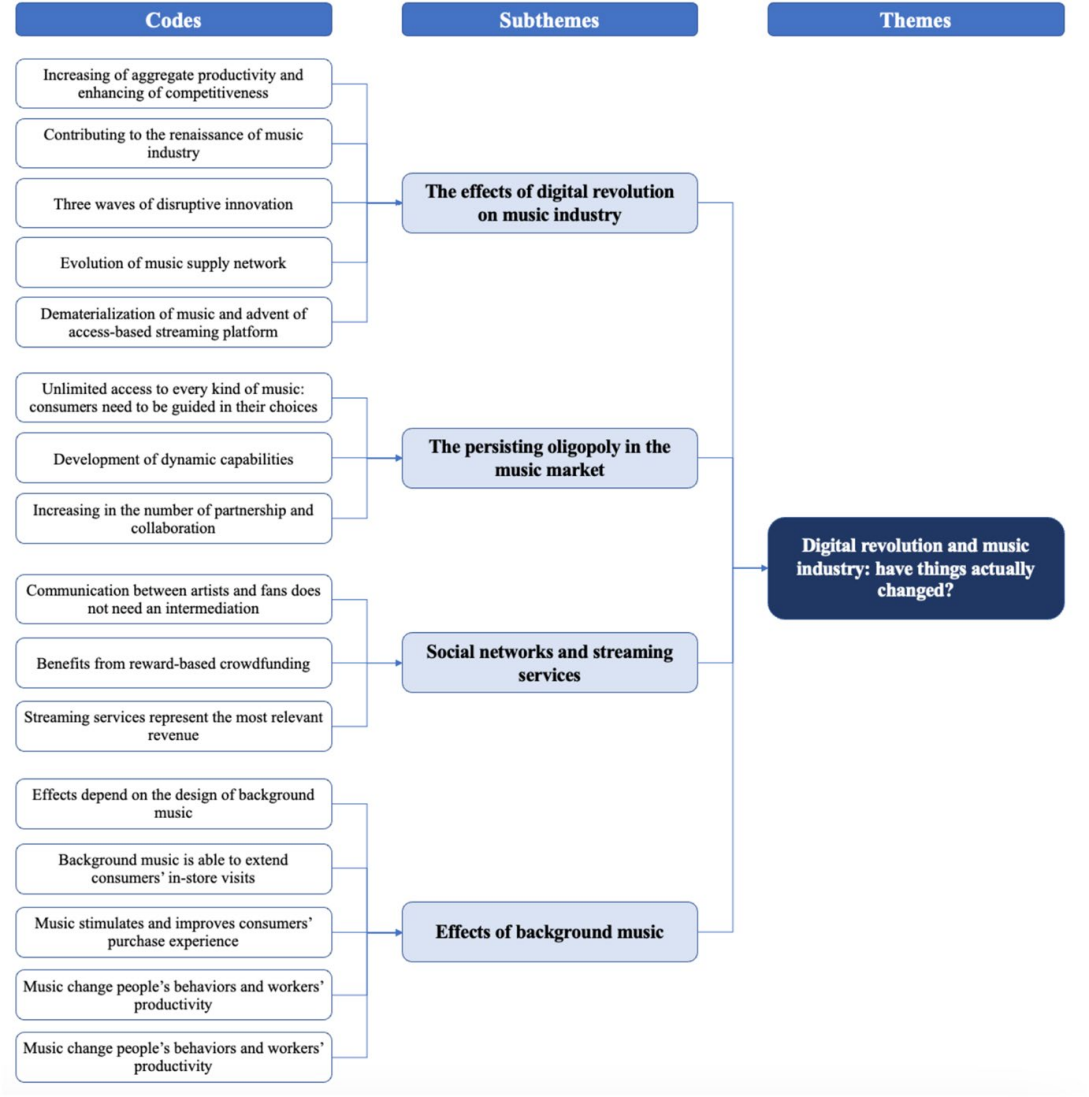

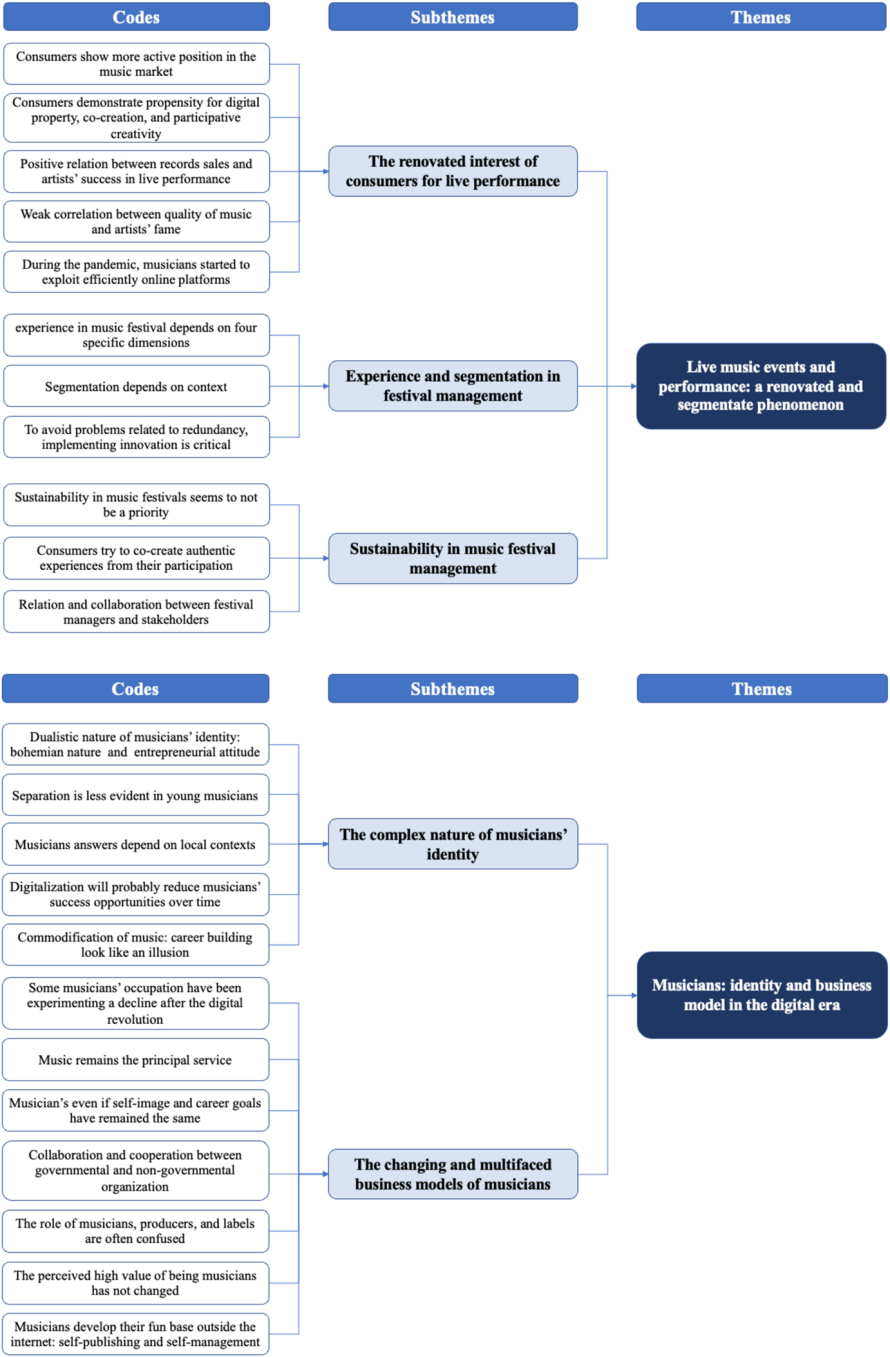
Codes, themes and subthemes emerged from selected literature

**Fig. 7:**
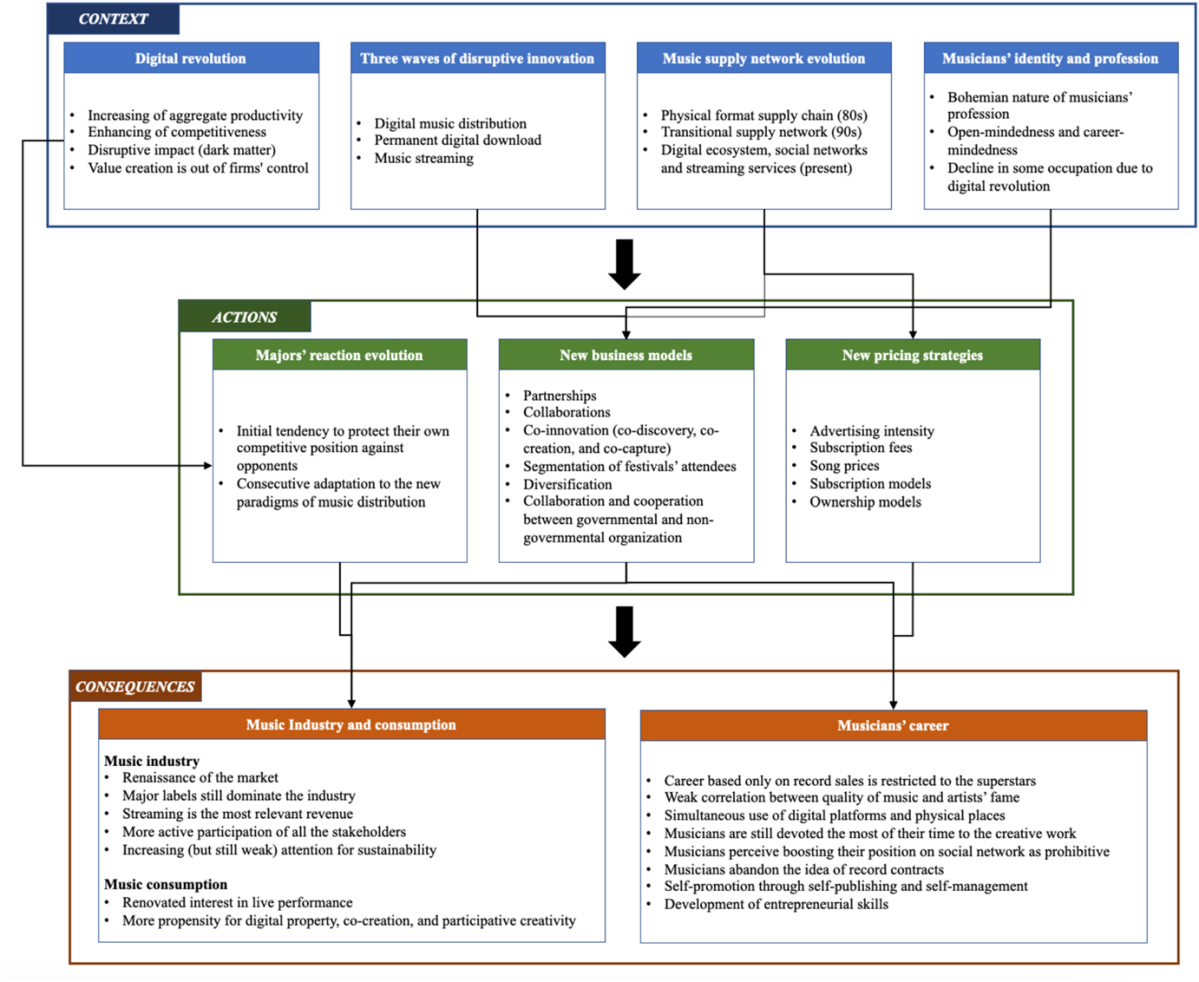
Conceptual map

This section presents the results obtained from the grounded analysis of the selected papers. Given the amount of data and the complexity of the concepts, two graphic representations have been created. Figure 6 shows a summary of codes, sub-themes, and themes that emerged from the analysis. Figure 7 shows a conceptual map of the field.

### The digital revolution and the music industry: Have things actually changed?

#### The effects of the digital revolution on the music industry

The digital revolution profoundly and directly impacted the economy through direct effects such as an increase in aggregate productivity and competitiveness and through more unobserved effects relating to the development of open-access platforms, new business models, and the need of managers to always achieve a better understanding of consumers’ expectations. These unobserved effects of the digital revolution have been described as digital dark matter (Vendrell-Herrero et al. 2017). Moreover, the inevitability of technology-related industry disruption seems not to be totally caught by professionals and firms, which are not reacting appropriately to this phenomenon (Riemer and Johnston 2019). For this reason, new business models are theorized in the literature. For example, Morrow (2018) introduced the concept of distributed agility as a model consisting of a reactive approach through which multiple self-organized teams combine their abilities to respond quickly to the rapid changes in the new digital market.

During the period 2011–2018, digital music and streaming contributed dramatically to the renaissance of the market (Nakano 2019). According to Nag (2017), the dramatic increase in the amount of accessible music contents and the decreasing barriers to consumer access is producing a contradictory effect on the philosophy of consumption. On one side is the benefit of freedom of choice and on the other side is an indirect call for more scarcity.

The literature has recognized three waves of disruptive innovations—digital music distribution, permanent digital downloads, and music streaming (Urbinati et al. 2019). Reactions to these things were similar; after an initial majors’ tendency to protect their own competitive position against opponents, there was adaptation to the new paradigms of music distribution. In particular, after the digital music distribution revolution, incumbents concentrated on building online shops and selling music from their catalogues. After the digital download revolution, incumbents verified the weaknesses in their previous strategic reactions and started to build relationships and partnerships with newcomers to fight the increased competition of important innovations such as Apple’s iTunes Music Store. Finally, with the music streaming revolution, entities such as the iTunes Music Store started to consider acquisitions as strategic choices to limit the damage caused by the rapid development of services like Spotify (Urbinati et al. 2019).

The music supply network evolved in a similar way. Nakano and Fleury (2017) identified three stages in this evolution. The first stage in the 1980s was a physical supply chain in which the hierarchical model with a vertical integration structure fostered the power of major labels that controlled technical and market access. The second stage in the 1990s was a transitional supply network in which the captive governance model fostered an open hierarchy. In this phase, the power was in the form of control over market access. Finally, in the last 20 years, the supply network evolved to a digital ecosystem in which individual producers and small and large providers can (at least theoretically) compete with major labels, which are exploiting their accumulated power to control mass market access through collaboration, partnerships, and new business models based on digital outlets and aggregators.

Sinclair and Tinson (2017) highlighted the problem of the decreasing value of psychological ownership of music guided by the dematerialization of music and the advent of access-based streaming platforms. Nevertheless, the antecedents of psychological ownership—investment of the self, profound knowledge and control of the target, and pride—demonstrated that consumers are modifying their experimentation with psychological ownership, achieving loyalty, empowerment, citizenship, and social rewards as consequences of this phenomenon. Therefore, through the new conceptualization of the psychological ownership of music, consumers are able to control the target of ownership and to develop and protect their music identity.

The other side of the coin of the digital revolution in music is that collaboration between major labels and other actors has allowed them to keep their power and to maintain the oligopoly in the market. In this context, while technological and digital innovation is stimulated, innovation in music is discouraged (Sun 2019).

The music industry has reacted to this tendency and has been able to assimilate the technological and digital innovations (Naveed et al. 2017). In particular, there are types of innovations that have assumed an increasing level of importance after the digital revolution. For example, co-innovation, a construct comprising co-discovery, co-creation, and co-capture, is widely recognized as a fundamental element in promoting correct governance, leadership, and resource integration (Saragih et al. 2019a, b).

#### The persisting oligopoly in the music market

The digital revolution and the digitalization of music produced an unexpected result in the music industry. Visionaries believed that the ability to achieve unlimited access to every kind of music—referred to as the celestial jukebox—would have led the music industry to overcome the oligopolist control of major labels and stimulate cultural diversity (Sun 2019). Nevertheless, the huge number of opportunities provided by the new digital market demonstrated that consumers need to be guided in their choices. The opportunities for musicians’ self-release apparently increased the degree of democratization of the music market. In fact, they consolidated the commodification and centralization of music whereby major labels still dominate the industry by promoting the conservative management of copyright ownership. This condition isolated self-released musicians, who have a marginal position in the industry. This is a worldwide condition, as verified in several articles, such as that by Qu et al. (2021). There is broad consensus regarding this condition and very few dissenters (e.g., Arbatani et al. 2018).

The digital revolution changed the pop-rock market, and streaming is now dominating the music industry, although there is still an important physical sector. Major labels have recently started a growth path after 15 years of crisis via the development of dynamic capabilities. In particular, technology allowed the emergence of non-hits and niches, increasing their opportunity to gain a long tail effect. Nevertheless, there is evidence of the persistence of the “superstar effect” (Coelho and Mendes 2019).

According to Prey et al. (2020), Spotify shows a bias towards major label content. Musicians’ success is dependent on services like Spotify. Therefore, Spotify’s preferences for major labels has caused discontent among minor labels and minor musicians.

Currently, the logic with which incumbents develop their strategy is correlated to the need for more timely approaches (Guichardaz et al. 2019). It is necessary to accept the competition of new technological innovations (e.g., iTunes and Spotify) and to develop suitable strategies to exploit the dynamic changes in the market (Trabucchi et al. 2017).

This evidence is confirmed in several studies. For example, Dellyana et al. (2017) state that the composition of actors in the music market has been changing over time. Conditions have become more dynamic, which stimulates the opportunity to increase the number of partnerships. Increasing the number of actors will change the general business model, allowing more opportunities for collaboration, including 360-degree, tailor-made partnerships; vertical integration; subscription services; pay per download; ad funded; bundles; and sponsorships. As a consequence, the many opportunities to exploit different sources of income leads actors in the industry market to develop new abilities and qualities. This modularity complicates the management of strategies and encourages the implementation of inter-firm alliances (Guichardaz et al. 2019). Through these collaborations, major labels are still dominating the music industry, even if independent labels and musicians can produce and distribute their music more easily and less expensively than before (Dellyana et al. 2017; Sun 2019). Major players like Spotify, Pandora, and Apple directly negotiate with the platforms to distribute artists’ music, while small labels must depend on intermediaries.

Some collaborations are, in fact, not official but extremely favorable. For example, the viral activity of fans on social media, such as YouTube, is a critical part of music stars’ strategy. Gamble et al. (2018) found that fans have an active role in the relationship between social media and the music industry. In particular, senior managers and music industry experts involved in the research argued that consumer-driven marketing campaigns can provide the best ideas regarding the execution, marketing, and managing of strategies. At the beginning of their careers, artists gain considerable advantages from social media and crowdfunding, even if fame comes as a result of their collaboration with major labels. Therefore, at the initial stage of their music success, the innovative nature of artists can be supported by fans’ activities, while at the advanced stage of their careers, artists tend to homogenize due to their involvement in the business logic of major labels business logics.

Nevertheless, evidence in the literature shows that collaborations and new business models are not the only way through which major labels react to the technological and digital changes in the market. Trabucchi et al. (2017) have shown that labels are able to re-invent old music solutions, such as CDs and vinyl, through innovations in their organizational structure and dynamic capabilities (e.g., changing their objective to entertainment enterprises), changing the meaning of their products from instruments for listening to music to collectors’ objects, and transforming the purchase process into an in-store experience. Therefore, developing dynamic capabilities seems to be the main response of major labels to the technological revolution in the music market. Changing the value proposition, capturing the new value of products, and customer orientation allow the major players to exploit the intersection between meaning and technology, thus modifying the competition among the incumbents.

#### Social networks and streaming services

Social networks have critical effects on artists’ success on online social network platforms. Communication with fun experimented a tremendous change after the digital revolution and the advent of social media. In the past, musicians used the press, television, and radio to attract fans’ interest. With social media and social networks, however, communication between artists and fans is direct. Communication with fans together with strategies that include the active sending of friend requests to fans or commenting on their posts can allow artists to control and influence their network (Ansari et al. 2018). Moreover, outside affiliations of artists can have a positive impact on the degree and density of networks (Ansari et al. 2018).

Social networks can have a dramatic effect on the consumption of music. For example, Spotify bundles music content in the form of playlists. This behavior allows Spotify to take over the market demand, reducing its dependency on major labels (Prey et al. 2020). Choi and Burnes (2017) studied the impact of using social media in the music industry and confirmed that social media have a considerable impact on fans’ engagement. Therefore, building solid and durable relationships and vitalizing participation should be considered a strategic focus for these firms. In the modern music market, firms cannot control value creation; therefore, they should use social media to enhance co-creation with fans, thus vitalizing their community and becoming the driving force in changing markets.

Business models in music benefit considerably from reward-based crowdfunding (Gamble et al. 2017), even though there is evidence suggesting that problems can arise due to young fans’ apprehension. Nevertheless, opportunities allowed by crowdfunding can help overcome limitations due to the need for constant consumer confidence. In fact, the development of partnerships with crowdfunding platforms has been increasing the opportunity for major labels to challenge the user-centric tendency of digital innovations (Gamble et al. 2017).

According to Arditi (2017), streaming services represent the most relevant revenue in the music industry since 2015. Streaming services are one of the most important reasons why the music industry recovered from the disaster of Napster and similar platforms for free music sharing. Indeed, streaming services are considered the (partial) solution to the decrease in music CD sales and to the increase in piracy. Moreover, there are similarities and differences between cultures about the future of streaming services. For example, Kim et al. (2017) show that US and Korean consumers prefer subscription-based on-demand streaming services and pay a higher price compared to the value of current streaming services. Nakano (2019) analyzed major US and UK outlets, recognizing that the preference of customers is for streaming services; however, there is still space for niches for which downloading is still preferred. Moreover, the existence of two major ecosystems, Android and iOS, has impeded the emergence of a dominant player to date.

Behavioral intentions connected to streaming service utilization depend on specific factors such as performance expectancy (i.e., the benefits for customers obtained by using streaming services), the degree of user-friendliness, social influence, the accessibility of resources and support, hedonic motivation, habit, and price (Barata and Coelho 2021). Suppliers of music services, such as streaming, should consider in-depth pricing strategies. Li et al. (2020) found that pricing strategies include advertising intensity, subscription fees, and song prices. Moreover, they considered subscription and ownership models and found that the first dominates the second only if advertisement revenues are limited. Therefore, an eventual mixed model turns to a subscription model when there are advertisement revenues, while it turns to an ownership model when revenues are higher.

The problem is to understand if the broad use of streaming services is a good thing for musicians as well. Musicians are losing control of the revenues that come from their music (Towse 2020). In fact, the opportunity to pursue a career based only on record sales is restricted to superstars. For example, in Norway, revenues originating from music copyrights represent only a small proportion of musicians’ earnings (about 20%). Therefore, the solution should be designed around live performance revenues. Nevertheless, the sustainability of streaming services in the long run is still debated in the literature and in professional practice.

#### Effects of background music

In-store background music produces positive and negative effects. The contradiction of scientific literature is illusory. In fact, such effects depend on the design of background music, which should adapt to the context and the service, which in turn is a critical moderator of the relationship between music and purchase intentions and customers’ in-store experience. For example, loud music has positive effects in retail settings but reduces customers’ experience in a bar. Classical, familiar, and highly recognizable music generally leads to positive effects. Nevertheless, in contexts in which they are inappropriate, the literature shows adverse consequences in terms of purchase intentions (Michel et al 2017).

Background music and creative support systems and their effects on consumers’ purchase intentions are fundamental to music literature (Michel et al. 2017). A great number of empirical studies have been undertaken to obtain improved understanding of such effects in every field. Music has positive and negative influences on consumers’ emotions within the environment in which they complete their purchases. Moreover, it is able to extend consumers’ in-store visits, promoting a name, brand, and experience that customers experiment with during their purchase process (e.g., Sassenberg et al. 2022). This is not limited to products but includes issues related to perceived images and positive or negative reactions to what consumers see. Klein et al. (2021) highlighted the existence of an inverse U-shaped relationship between the complexity of images and the level of appreciation that music is able to modify, promoting a better reaction to simple images. In other words, music can move consumers’ attention from complex to simple stimuli. For this reason, music can be used with simple images to stimulate and improve consumers’ purchasing experience.

Background music can be seen as a functional instrument for social control because it can change people’s behaviors and workers’ productivity, although its collateral effects are still unpredictable (Karakayali and Alpertan 2020). Indeed, music is a mediator of emotions and attitudes; it can modify performance and generate a significant effect in the workplace, although further in-depth analyses are needed to clarify its effects (Landay and Harms 2019). An important review by Landay and Harms (2019) highlighted that the relationship between the music heard and mood and negative and positive emotions is moderated by extraversion (i.e., the tendency to concentrate on gratification from outside individuals), task complexity, and listening autonomy. Moreover, they found that the link between listening to music and task performance is highly contextual and depends on “the availability of cognitive resources and the type of task” (Landay and Harms 2019, p 379). The evidence shows that workers with more experience in selecting music for personal listening can show greater improvement in their task performance.

### Live music events and performance: a renovated and segmentate phenomenon

#### The renovated interest of consumers in live performance

The co-evolution of streaming and live music has had positive consequences for the music industry (Naveed et al. 2017). It allowed the recovery of the music industry and the more active participation of all stakeholders. Moreover, consumers have a more active position in the music market, demonstrating more propensity to acquire digital property, for co-creation, and for participative creativity (Naveed et al. 2017). In particular, consumers contribute to the creation of value even during the phases of product development and commercialization (Saragih 2019). In the new digitalized era in which musicians’ control of their records sales is weaker, live performance is assuming increasing importance.

In general, the analyzed literature seems to agree on consumers’ renovated interest in live performance. For example, Papies and Van Heerde (2017) confirm the positive relationship between artists’ success in records sales and their success in live performance. Nevertheless, the authors verified that the opposite relationship is weaker, as it is moderated by piracy and unbundling, even if piracy seems to be a decreasing trend in some important markets (e.g., Germany). Moreover, they found a weak correlation between quality of music and artists’ fame. Nevertheless, the evidence shows that this condition of renaissance is not common to all music genres. Pompe et al. (2017) found that classical music is experiencing a crisis, mainly because consumers say they do not have enough time to enjoy classical concerts. Therefore, the problem of how to add value to some niches is still being debated in the literature.

During the period analyzed in this literature review, Covid-19 impacted the world. In this context, quarantines obliged musicians to be part of lockdowns. Concerts and live music events were cancelled, and musicians had to face a new and challenging reality. However, some positive consequences emerged. In fact, the research showed how during the pandemic musicians started to efficiently exploit online platforms to share their music and to organize 100% online music events (e.g., Areiza-Padilla and Galindo-Becerra 2022). Therefore, in the future, digital platforms should be used simultaneously with physical places to share the experience of live events, capturing a wider audience and the attention of a greater number of potential consumers.

#### Experience and segmentation in festival management

Aşan et al. (2020) found that experience in music festivals depends on four specific dimensions—aesthetics, which refers to tourists’ evaluation of festivals’ physical environment; entertainment, that is, the active or passive participation of tourists in watching shows; the opportunity to escape daily life, become immersed in a different context, and live a separate experience; and education, that is, gaining new skills and knowledge from the experience of participation. The four dimensions contribute to the formation of participants’ perceived value of the festival, which is a significant mediator between experience and satisfaction. The general conditions in which festivals are organized are often complex, spontaneous, and sometimes unexpected (Laberschek et al. 2020). Therefore, going beyond these general dimensions is critical. Other factors should be considered to increase the value promoted by a specific music festival. Because festivals are often devoted to a particular music or art genre, segmentation is one of the most critical elements of managerial strategies.

The segmentation of music festival attendees is considered to be extremely dependent on context (Kim and Kang 2022). For example, Kinnunen et al. (2018) studied Finnish rhythm music festival audiences and identified three segments—omnivores and the loyal heavy tribe, which represent the oldest attendees, and the hedonistic dance crowd, which represents the youngest attendees. Mallette et al. (2018) discussed the niche example of military music festivals. The authors found two main dimensions of audience segmentation—the appeal of the event and the degree of affinity—that produce different levels of motivation to participate. Roll et al. (2014) showed the importance of place and the medium of live operas, as they are meaningful for attendees. In particular, consumers consider opera from a holistic viewpoint. Therefore, brand communication should include the fact that the meaning of results differ depending on the samples. There are examples of specific techniques of segmentation. For example, Kruger and Viljoen (2021) use psychographic segmentation to identify three distinct segments of attendees. They considered motives for attending, behavioral intentions, and global causes aimed at eradicating poverty. The research shows the relevance of segmentation in valorizing the nature, objects, and goals of music festivals.

Modern music festivals have a more general background. Therefore, attendees are of various natures and have different music preferences. Consequently, to avoid problems related to the redundancy of festivals’ organization and commercial proposals, implementing innovation is critical. Li et al. (2017) identified a strategic factor for success in implementing innovation. In particular, they distinguished between stakeholders’ satisfaction, achieved through harmonious relationships, and process efficiency, achieved through functional relationships. The greater the extent of both relational characteristics (i.e., harmony and functionality), the greater the opportunity to achieve successful innovation implementation.

#### Sustainability in music festival management

Sustainability in festival and event management is closely related to the three traditional dimensions—economic, social, and environmental. Some studies have been devoted to sustainability management to achieve a better understanding of the actual interest of managers in the best practices for fostering the sustainable management of events. For example, Wickham et al. (2021) performed a qualitative analysis of 10 international music events and identified 14 best practices connected to sustainability. Economic sustainability was associated with attracting financial capital, artists, and performers; maintaining permanent management; and the reporting of event-related benefits. Social sustainability was associated with attracting experts and influential educators; the supply chain; and the reporting of event-related social benefits. Finally, environmental sustainability was associated with attendees and the behavior of supply chain partners.

At the same time, the research has clarified that the benefits connected to music festivals (e.g., financial and social benefits) are overestimated by participants and urban communities. Nevertheless, eventual negative effects, such as pollution, parking difficulties, and traffic congestion, are often anticipated and resolved by festival promoters (Han et al. 2017).

However, sustainability seems not to be a priority of music festivals. Dodds et al. (2020) found that 64% of Canadian music festivals included in their sample did not communicate about their sustainability practices. Moreover, only 6% of them concentrated their sustainability campaign on social media, even though the literature shows that active communication is critical for developing and maintaining good relationships with participants (Luonila and Kinnunen 2019). Contemporary music festivals have critical socio-spatial consequences and meanings; consumers try to co-create authentic experiences from their participation, even though the nature of such experiences can be considered a commercial imperative (Szmigin et al. 2017). Co-creation was connected to the sustainability of festivals in the work of Werner et al. (2019), who identified three categories of festivals attendees—the sustainable co-creation type, focused on the creation of an altruistic environment; the experience co-creation type, focused on the contradiction between real life and the experience of festivals; and the calculating co-creation type, who weight the processes of giving and acquiring value from the experience of festivals. In this sense, the benefits associated with music festivals are co-created by both attendees and organizers. Therefore, identifying the most relevant stakeholders is critical for the diffusion of the idea of festival sustainability.

Relationships and collaboration between festival managers and stakeholders, including the attendees’ perspectives, can foster the incorporation of sustainability practices. According to Hazel and Mason (2020), if sustainability is defined as a core value of music festivals, forms of collaboration such as sponsorship contracts can encourage the development of relationships among stakeholders who share the same sustainable values. Therefore, identifying the right stakeholders can bolster festivals’ financial and cultural components, as well help integrate the original value of festivals and sustainability needs (Hazel and Mason 2020; Richardson 2018).

### Musicians: identity and business models in the digital era

#### The complex nature of musicians’ identity

The identity of musicians in the modern competitive music industry has been widely discussed in the literature. Schediwy et al. (2018) identified the dualistic nature of the scientific debate. On one hand, there is a considerable number of contributions that glorify the bohemian nature of the music profession. On the other hand, other papers recognize that assuming an entrepreneurial attitude is an unavoidable need for musicians. Moreover, keeping a stable identity is challenging for musicians, due to the instability of income, uncertainty, and exploitative tendencies in the market.

Schediwy et al. (2018) found that the separation between the bohemian and the entrepreneurial identity of musicians is less evident in young musicians. In particular, two factors contribute to the formation of musicians’ identity, open-mindedness and career-mindedness, which combine the bohemian nature of the music profession with the necessary market orientation.

The relationship between musicians’ artistic and entrepreneurial identity has been recently studied by Pizzolitto (2021). His research revealed the profound dilemma between musicians’ traditional view of the art and the necessity to change and adapt their business models depending on the economic conditions. Everts and Haynes (2021) studied the contexts of the British and Dutch music markets and found that musicians’ entrepreneurial identity is based on the rapid change of musical contexts and local contexts. The Netherlands has been building an institutionalized pathway to let musicians’ artistic needs meet their entrepreneurial sensibility. Nevertheless, according to their research, the conditions that musicians have to face in pursuing their music careers are extremely complex. For example, opportunities are inversely proportional to the number of young aspiring musicians; only a small proportion of musicians achieve a successful career; and digitalization, which seemed to be a possible way to increase the number of successful independent musicians, will probably reduce their opportunities over time.

This tendency is quite common in the world. Güven (2020) considered the music environment in Turkey and found that musicians there are experiencing increasing difficulties in their creative work. In fact, they do not talk about solidarity in the music world. Instead, they talk of the increasing commodification of music where solid experience and career building seem to be an illusion. Therefore, we have to ask why there are always more young people trying to establish a career in music (Everts and Haynes 2021).

#### The changing and multifaced business models of musicians

Musicians need to diversify their business models by composing, producing, and distributing their music. Given the degree of technological evolution, understanding the future of independent musicians’ business models is complicated. A considerable number of musicians have experienced a decline after the digital revolution in terms of professional studio performance and recording (Herbst and Albrecht 2018). Nevertheless, in this framework, music is the principal service in a portfolio of services entirely managed by musicians (Eiriz and Leite 2017). Therefore, even if a musician’s self-image and career goals remained the same before and after the digital revolution, there is a general consensus in the music market regarding the fact that the way to achieve career objectives has changed (Schwetter 2016).

In new business models, collaboration and cooperation between governmental and non-governmental organizations, among different kinds of art and culture, and among artists seems to guarantee partial independence from record contracts (Ibrahimova 2019). For example, in the hip hop environment, Carter and Welsh (2018) found that collaboration among rappers increases their visibility. Among hip hop artists, there is a tendency to abandon record contracts to pursue a solitary career. A specific example is Alessandro Aleotti (alias J Ax) in Italy who in 2013 decided to quit his contract with New Sound and start his own record label called Newtopia with another famous Italian hip hop artist, Federico Leonard Lucia (alias Fedez).

The choices of independent musicians and labels about where and what to record are extremely contextual and related to the need for non-traditional recording locations (Walzer 2016). The role of musicians, producers, and labels are often linked and therefore confused. Consequently, the quantification of their contribution to the production of independent music is complex. Nevertheless, the evidence shows that producers’, musicians’, and sound engineers’ contributions are critical for communication during the decision-making process (Walzer 2016).

In summary, the evidence in the literature underlines the complexity of the music market for musicians. This precondition could lead to the reasonable conclusion that musicians should dedicate much more time to the commercial side of their work. Nevertheless, and counterintuitively, publications show that musicians still devote most of their time to creative work. Everts et al. (2021) studied the early career experiences of Dutch musicians and found that the strong dynamicity of the music industry does not change the perceived high value of being musicians. In fact, musicians do not spend their time trying to boost their position on social networks because they perceive that the funds needed to flourish in that environment are becoming prohibitive. Therefore, they devote their time to creation and to the development of their fan base outside the internet. Finally, they show to enjoy the entrepreneurial part of their activity.

Consequently, the digital revolution, together with the persisting situation of oligopoly in the market, allowed musicians to abandon the idea of record contracts and to embrace self-promotion through self-publishing and self-management (Schwetter 2016). The instability of direct revenue experienced by independent musicians and labels resulted in a substantial change after the digital revolution. The digitalization of music had a dramatic impact on musicians’ activity, particularly for independent musicians who are becoming more than composers and who are developing entrepreneurial skills that were traditionally the purview of labels and agencies (Eiriz and Leite 2017). Moreover, laws and regulation for the management of copyright have been designed to improve consumers’ interests and rights and show weakness in the connection between copyright and the collective needs of management organizations, thus resulting in an unfair advantage for major labels (Schroff and Street 2017).

## Future research opportunities

Based on the content analysis in the previous section, multiple future research opportunities can be identified. These opportunities emerge with the same distribution as the themes and subthemes from the grounded theory analysis. Table 1 presents a summary of the most relevant research questions that have been deduced from the articles’ contents.

**Table 1:** Future research opportunities

Themes	Research question	Recommended methodology
*The digital revolution and the music industry: Have things actually changed?*		
The effects of the digital revolution on the music industry	What is the relative role of technology in the recovery of the music industry after the previous periods of stagnation?	Empirical
The effects of the digital revolution on the music industry	What is the relative role of new forms of financing in the recovery of the music industry from the previous periods of stagnation?	Empirical
The persisting oligopoly in the music market	What are the non-financial variables that allow major labels to maintain their leadership positions in the music market?	Theoretical, empirical
The persisting oligopoly in the music market	What are the commonalities and differences in the competition between major and minor labels and artists?	Empirical
Social networks and streaming services	What are the most critical variables connecting consumers and musicians after the digital revolution?	Theoretical
*Live music events and performance: A renovated and segmentate phenomenon*		
The renovated interest of consumers for live performance	What are the best strategies to engage consumers with the concept of “meaning”?	Theoretical
Experience and segmentation in festival management	What are the connections between chaos theory and the management of events such as festivals?	Theoretical
Experience and segmentation in festival management	What are the theoretical foundations of the internationalization of niche music festivals?	Theoretical
Experience and segmentation in festival management	Can futuristic management philosophies, such as holism, post co-creativism, and deterministic chaos theory, be applied to festival management?	Theoretical
Experience and segmentation in festival management	What are the cultural elements that increase consumers’ tendency to avoid traditional purchasing methods?	Empirical
Experience and segmentation in festival management	What effects do differentiation strategies have on consumers’ expectations about hedonic music performances?	Empirical
Sustainability in music festival management	How can local music and record stores organize experiential contents to overcome the current status of their crisis?	Theoretical
Sustainability in music festival management	What are the positive effects on consumers’ experiences and profits resulting from the application of sustainability management practices?	Empirical
*Musicians: Identity and business models in the digital era*		
The complex nature of musicians’ identities	What psychological statuses allow musicians to accept their entrepreneurial nature?	Empirical
The complex nature of musicians’ identities	What historical evidence can be found of the intersection between the music profession and entrepreneurship?	Empirical
The changing and multifaced business models of musicians	What is the future of management for hybrid products preserving cultural heritage and traditional music?	Theoretical, empirical
The changing and multifaced business models of musicians	What are the most effective and financially sustainable products for preserving traditional music?	Theoretical, empirical
The changing and multifaced business models of musicians	What are the best and most affordable uses of technology for enhancing the efficiency of strategies for minor labels and artists?	Theoretical, empirical
The changing and multifaced business models of musicians	How can technology be organized to improve the opportunities for minor artists and independent labels to be found by consumers’ research using existing technological instruments?	Theoretical
The changing and multifaced business models of musicians	What are the elements that allow minor artists to emerge without having access to major artists’ financial and power-related assets?	Empirical
The changing and multifaced business models of musicians	What are the mediators and moderators of consumers’ opportunities to satisfy their music needs via alternative music and independent artists?	Empirical

Concerning the role of digitalization in the revolution of the music market, the analysis showed that it contributed to recovering from the previous period of stagnation (e.g., Dellyana et al. 2017). Therefore, future research can examine the contribution of specific factors that may or may not be connected with the technological revolution in more depth. In particular, it can concentrate more on the new forms of financing (e.g., crowdfunding) and their influence on the growth of the music industry. Moreover, researchers can perform in-depth analysis of the determinants of the major labels’ ability to maintain their leadership position after the digital revolution (Guichardaz et al. 2019). In particular, researchers can concentrate on the effect of a set of variables that exclude financial logic. Furthermore, as this market condition allows two different levels of competition, researchers could focus on the commonalities and differences that can emerge from the study of this dualism.

Trabucchi et al. (2017) reflected on the opportunity for artists and labels to base their competition strategies on the concept of “meaning.” More conceptual papers on this topic should be published to allow music actors to embrace new strategies connected to this concept. In particular, theoretical articles could help artists and labels better connect their visions, missions, and brands in a practical execution of “meaning” for their products.

Concerning the position of major labels and their permanent leadership position in the market, research on this is based on consumers’ expectations about hedonic performances (Chen et al. 2018). In particular, the logic of differentiation could reduce the distance between the two levels of competition that emerged in the music market. Therefore, more research on single case studies is needed to understand what elements allow minor artists to emerge without having access to major artists’ financial and power-related assets. Specifically, research should concentrate on the conditions that allow the reduction or elimination of barriers that traditionally impede the emergence of minor artists and labels (e.g., Pillai et al. 2021; Walzer 2016). Finally, researchers should help minor labels and artists find better applications of existing technologies characterized by low costs.

One of the positive effects of the digital revolution is that the available technology allows artists and producers to be more creative in their work. Therefore, new variables have started to affect the basis of consumers’ access to music listening. Even if research has found some interesting connections between critical variables (e.g., advertising and reservation; Li et al. 2020) and the new forms of consumer access to music listening, more empirical research and case studies are needed to fully understand this phenomenon. Moreover, technology has made consumer research related to music easier and more efficient. Nevertheless, it seems that this condition has not changed the general competition in the music market. Therefore, more empirical research is needed to understand the mediators and moderators of consumers’ opportunities to satisfy their music needs via alternative music and independent artists. In other words, research should study new methods for improving the opportunities for minor artists and independent labels using the existing technological instruments.

Concerning the negative effects of the digital revolution on the music markets, research has focused on the complex conditions of local record and music stores. In this regard, more conceptual papers are needed to evaluate new forms of experiential content through which local record stores can overcome the current crisis. Moreover, empirical analyses should be performed to obtain a deeper understanding of the cultural elements that increase consumers’ tendency to avoid traditional purchasing methods.

Multiple papers have debated the role of music festivals in business and management studies. Music festivals are widely recognized as complex phenomena in which the management instruments needed to achieve results should be organized and planned using holistic and innovative logic (Laberschek et al. 2020). Empirical research in this field has been sufficiently developed, and the literature indirectly calls for more conceptual studies. Specifically, it seems that in this field, practice is more advanced and faster compared with theory. Therefore, theoretical research should concentrate on multidisciplinary issues to ensure that festival managers have opportunities to build their style while drawing on solid and consolidated theoretical foundations; this will limit the chaotic and unpredictable events occurring in festivals at present.

First, the theoretical papers concentrate on the connection between chaos theory and management, applying these connections to music festival management. It is necessary to theorize new advancements in chaos management before they are applied in practice. Music festival management needs to refer to innovative and challenging management theory to develop new methods of obtaining economic, financial, social, and sustainable results from the event organization.

Second, conceptual research should be performed to increase the understanding of elements that improve consumers’ experience during festivals. Theorizing about management methods for niche festivals in which a specific kind of music is considered can help organizations stimulate consumers’ experience. In particular, theoretical studies of the foundation of the internationalization of festivals referring specifically to niche festivals can be critical for finding solutions to management issues (da Cunha Brandão and Oliveira 2019).

Third, the literature firmly calls for a better understanding of the conceptual foundations of the concept of value in music festivals. Holism, post-co-creativism, and the control of socioeconomic turbulence and chaotic issues related to the management of festivals are widely recognized as powerful instruments for value creation (e.g., Gozini and Tseane-Gumbi 2017; Robertson et al. 2018). Moreover, co-creation and co-innovation seem to play a critical role in the panorama of actors involved in these events. Similar practical consideration represents a strong call for pushing conceptual analyses to a higher level; in this way, management theory can overcome the limitations of being directly connected to managerial practices and can achieve a superior stage of abstraction.

In addition to the need for theoretical research, more empirical research is needed on the role of the music market in sustainability issues. There is still a distance between the sincere interest in sustainability management and its realization (e.g., Richardson 2019; Raffay-Danyi and Formadi 2022). Therefore, empirical research should consider single and multiple case studies concerning the positive effect on consumers’ experience and the benefits resulting from the application of sustainability management practices. A case study should be performed using longitudinal logic to highlight differences in terms of praxis and results and to guide future management when it comes to investing time and resources in implementing such logic efficiently.

Finally, the literature revealed considerable interest in the dualism between music and profits. The problem of musicians being reluctant to be considered entrepreneurs has been studied in a number of empirical publications. A possible promising lens for analysis could come from the psychological theory of professional identity (e.g., Marcia 1966). In particular, the literature could benefit from more qualitative research based on in-depth interviews and surveys analyzing the status of musicians’ identities in depth. The final aim of this research is to understand what identity status can lead musicians to overcome the obstacle of their reluctance to consider music in the same manner as all other fields. In this sense, more historical research could be beneficial to overcome the dualism between art and entrepreneurship. Harbor (2020) demonstrated that a considerable number of marketing interventions were used during the seventeenth and eighteenth centuries to promote music concerts and the arts. More historical references to the connection between entrepreneurship and the arts could be helpful in overcoming this problematic dualism.

## Discussion and managerial implications

In the last decade, the music market has been greatly affected by the digital revolution, and technology has caused profound internal changes. This revolution has increased the opportunity for the diffusion of music through several services, means, and platforms, even though the oligopoly of major labels has continued to dominate the market. Nevertheless, the exploitation of these new opportunities is limited by the high level of financial and technical resources needed to access innovative technologies. Therefore, although the digital revolution has increased opportunities for minor artists and labels, the competition is still divided into two levels—the oligopoly of major labels and artists and the super competition of minor labels and artists. To overcome this limitation, futuristic strategies need to be conceptualized and operationalized, and most probably they will be based on the concept of “meaning” (Trabucchi et al. 2017).

Papies and van Heerde (2017) identified concerts as one of the most powerful instruments through which minor artists can improve their opportunities to flourish. The empirical literature concentrates on understanding the effects of specific management styles on the organization of events and festivals. Therefore, there is evidence of the fundamental role that concerts and live events will play in the future. Events are seen as chaotic and complex phenomena in which managerial styles cannot be centered on the organization but have to be based on co-creation and co-innovation, promoting revolutionary organization initiatives to enhance opportunities for minor artists and labels to emerge and succeed. Co-creation and co-innovation should be achieved through the involvement of musicians, record labels, and consumers, resulting in a mutually useful network in which access to high levels of financial resources is not an issue. In this sense, the philosophy through which musicians should interpret their role in society has to change in order to promote their role as self-entrepreneurs and to develop their business identity.

This review led to the identification of numerous managerial implications, mainly concentrated on event and store management, sustainable management logic, and the condition of musicians in the market. In terms of improving consumer engagement, managers should place more emphasis on consumers’ preferences than on the cost of performance (Kim et al. 2022). The literature highlights that consumers are still interested in the principal product of the music market—*music* (Papies and van Heerde 2017). In this sense, particular attention has to be paid to music festivals and events. During festivals, the aesthetic of the offering has been recognized as a fundamental factor in achieving the events’ objectives. Furthermore, surprising consumers with unique stimuli is critical to increase their engagement (Loureiro et al. 2021). In fact, organizations should challenge the status quo by collecting as much information as possible on the changing nature of their audiences. Hiring a community manager who conducts research on social media and promotes events depending on the participants’ preferences can be a strategic factor in improving the quality of integrated communication between organizations and consumers and in increasing loyalty among attendees (Llopis-Amorós et al. 2018). Moreover, managers should go beyond adapting their organizations to the changes related to social media platforms and should build solid relationships with their customers, thus increasing the opportunity to achieve greater loyalty and improving their opportunity to involve consumers in the process of co-creation (Choi and Burnes 2017).

The literature discusses sustainable management logic. In general, overcoming the traditional barriers and roles in management logic seems to be fundamental for the future of marketing strategies in this field (Vendrell-Herrero 2018). In particular, Kullak et al. (2021) suggested that managers of minor organizations should develop and coordinate a network in which actors have access to relevant resources. Moreover, as there is evidence about the difficult conditions of advertisement-related returns, music suppliers should differentiate their policies depending on this variable. Li et al. (2020) recommended that managers should choose an ownership, subscription, or mixed-pricing model according to their level of advertisement returns.

Sustainable management in music should be achieved through improving communication between suppliers and customers. In particular, it is perceived as critical to develop technological tools to allow for the establishment and maintenance of a relationship between distributors and consumers (Tran et al. 2018). In this sense, the literature recognizes that the technological revolution is one of the most important opportunities for music managers to change their organizational logic. Technology “favour[s] smaller, more flexible companies that are more conducive to innovation” (Renard and Hallam 2018, p 182). Firms should put their traditional models and decision-making procedures under review, concentrating on changing definition of “meaning” that music consumers adopt over time (Trabucchi et al. 2017).

The problem of sustainability has also been analyzed in light of the difficulties that small music enterprises face when it comes to accessing high levels of financial support. For example, Andersén et al. (2019) showed that to improve their growth opportunities, small firms can exploit their relationships with environmental-oriented suppliers and their ability to develop green purchasing capabilities. The sustainable management logic also relates to an improvement in the congruency between in-store background music and the purchase experience that sellers want their customers to have. The literature highlights the importance of the correlation between advertising, music, and all other stimuli and environments in which purchasing takes place (Michel et al. 2017). In fact, the correct combination of music and advertising can be useful for changing brand perceptions. For example, an intense sound and low pitch can communicate masculinity, whereas high-pitched sounds can communicate femininity (Zoghaib 2019). Moreover, empirical evidence has shown that major tones and faster tempos improve the purchase experience (Liu et al. 2022). Music associated with different levels of arousal can be used to manipulate consumers’ feelings; for instance, uncomfortable situations can be smoothed out through background music characterized by low levels of arousal (Roy and Das 2022). Stable tonal structures can induce cheerfulness, whereas unstable tonal structures communicate sadness (Zoghaib 2019). Therefore, if sellers want to prolong consumers’ purchasing experiences and in-store visits, they should choose music of a low tempo and volume (Michel et al. 2017).

The literature discusses the position of musicians in the music business. In particular, various articles debate the opportunities that music allows professionals depending on their level of popularity. For example, Papies and van Heerde (2017) observed that concerts are critical for both famous musicians and musicians who are not famous. For famous musicians, concerts represent an opportunity to consolidate their position in the market. For less famous artists, concerts should be considered marketing strategies to increase their popularity, that is, as launching pads for their future careers.

## Conclusions

Music comprises a dynamic, complex, and chaotic environment in which futuristic management styles and co-creation, co-innovation, and post co-creation logics should be considered in planning and operationalizing strategies at every level of competition. Although the digital revolution has transformed many aspects of the music business and management, several issues continue to limit its evolution. This SLR clarified that in the future, a considerably relevant role will be played by events, festivals, and concerts whereby innovative managerial styles can overcome the complex conditions of minor artists and labels and allow them to flourish. Nevertheless, the picture of music generated from the literature is still evolving. The future of the field seems to demand higher levels of philosophy around business issues and management styles through which obstacles relating to the position of musicians as entrepreneurs will be overcome, and products will be considered for their meaning rather than for their cost.

## Data Availability

The datasets generated during and/or analysed during the current study are available from the corresponding author on reasonable request.
